# Diabetic ulcer with diabetic neuropathy: a rare clinical image

**DOI:** 10.11604/pamj.2022.42.127.35369

**Published:** 2022-06-16

**Authors:** Kapil Sharma, Swarupa Chakole

**Affiliations:** 1 Department of Medicine, Jawaharlal Nehru Medical College, Datta Meghe Institute of Medical Sciences, Sawangi, Wardha, Maharashtra, India,; 2 Department of Community Medicine, Jawaharlal Nehru Medical College, Datta Meghe Institute of Medical Sciences, Sawangi, Wardha, Maharashtra, India

**Keywords:** Diabetic ulcer, neuropathy, demyelination

## Image in medicine

A diabetic ulcer is a non-healing ulcer seen in people with uncontrolled blood sugar. High blood sugar causes the formation of sorbitol from sugar, which causes demyelination of large nerve fibers. Here we reported a case of a 62-year-old male who came to the medicine outpatient department with the complaint of a large ulcer over the anterior aspect of the right leg. In diabetic patients, there is an altered immune system and poor blood flow. On physical examination, there is loss of epithelium with granulation tissue formation and decreased sensation, doctors suggested a diagnosis of diabetic ulcer with diabetic neuropathy. On lab investigation, the patient was diagnosed with type II diabetes with an uncontrolled blood sugar of chronic duration.

**Figure 1:**
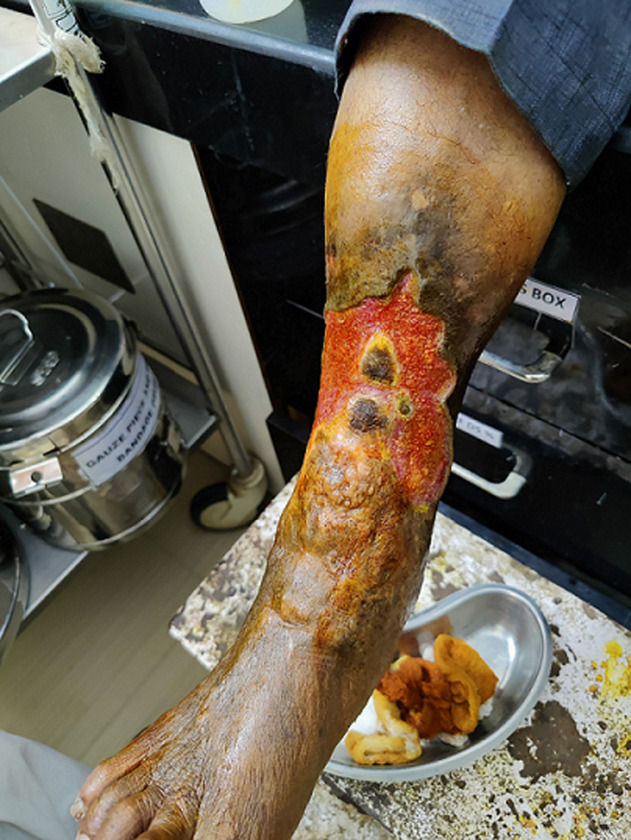
loss of epithelium with granulation tissue formation

